# When Different Language Groups Meet Online: Covert and Overt Focus on Form in Text-Based Chats

**DOI:** 10.3389/fpsyg.2021.739543

**Published:** 2021-09-22

**Authors:** Ruiling Feng, Kyunghee Pyun, Wenzhong Zhang, Rafael Márquez Flores

**Affiliations:** ^1^Foreign Language College, Tianjin Normal University, Tianjin, China; ^2^Department of Foreign Languages and Literatures, School of Humanities, Tsinghua University, Beijing, China; ^3^Department of History of Art, School of Liberal Arts, Fashion Institute of Technology, New York, NY, United States; ^4^College of Foreign Languages, Nankai University, Tianjin, China; ^5^Languages, Teaching, and Cultural Diversity Major, Universidad Popular Autónoma del Estado de Puebla, Puebla, Mexico

**Keywords:** focus on form, covert, overt, text-based online group chats, collaborative online international learning, EHL-EFL group, EFL-EFL group

## Abstract

Focus on form has been extensively studied in text-based online dyadic chats but much less has been explored in group chats with interlocutors from different language backgrounds. Additionally, there are very few studies investigating covert focus on form. This study investigated the effects of interlocutor types on errors and focus on form episodes, both covert and overt, in text-based online group chats. We collected chat logs from two collaborative online international learning projects. One project was developed for the collaboration between an English course at a Chinese university and an art history course at a U.S. university; the other between another cohort of the same English course and a cultural studies course at a Mexican university. We compared errors, feedback, and other characteristics of focus on form episodes between the two projects. Analyses revealed significant differences in characteristics such as overtness (overt, covert), linguistic focus (mechanical, lexical, and grammatical), and source (code, message). However, no significant differences were found for the type of focus on form (preemptive, reactive), presence of uptake, uptake quality (successful, unsuccessful), and repair provider (self, other). Students showed a preference for self-repair over other-repair and for lexical focus over mechanical and grammatical foci in both projects. Overall, only a small proportion of errors were followed by feedback. Therefore, a small amount of uptake and successful uptake occurred in both projects. The results can shed light on how instructors could provide effective scaffolding and tasks to make virtual exchange projects more rewarding.

## Introduction

Focus on form (FonF) refers to the treatment of linguistic elements incidentally arising in meaning-focused communication (e.g., Long, 1991; Doughty and Williams, 1998; Ellis, 2001, 2016). Lyster and Ranta (1997) examined reactive FonF initiated with an error. Ellis et al. (2001) expanded the concept to include preemptive FonF instigated with a query instead of an error. FonF in text-based online chats might be overt that can be retrieved from the chat history alone or covert when interlocutors try to fill language gaps with resources such as dictionaries, online translation tools, or people physically around them (Smith, 2010). Covert FonF in this study includes but is not limited to self-initiated self-repair moves before sending a message out (Smith, 2008, 2010); it also refers to noticing without negotiation with another interlocutor (Peace, 2019).

An overt focus on form episode (FFE) generally includes feedback and possibly uptake. Feedback could help notice language gaps between one's interlanguage and the target language (Loewen and Sato, 2018; Sato and Mcdonough, 2020). Panova and Lyster (2002) found lexical focus more common than other foci in feedback in face-to-face communication. Smith (2003) and Kwon and Lee (2011) reported similar findings in text-based online communication. Feedback is scarce in meaning-focused communication because of either overlooking errors or refraining from offering feedback owing to social and affective factors (Ballinger, 2015; Peace, 2019). Uptake was first defined as what learners report they have learned (Allwright, 1984) and later as immediate learner responses to feedback (Lyster and Ranta, 1997). Uptake with more target-like modified output (pushed output, Swain, 1995, 2000) could contribute to interlanguage development (Loewen, 2005).

Research gaps still exist in studies of FonF in text-based online chats. First, covert FonF has drawn little attention. Language learners often stop short of expressing themselves (Hanaoka and Izumi, 2012), fail to understand others, or overlook errors in previous messages (Izumi, 2013; de Vos et al., 2019). These language gaps might be filled more often with covert FonF than with overt FonF in text-based online group chats. Second, most FonF studies of text-based online chats examine dyadic interaction, typically between a native speaker and a learner or between learners from the same language background (e.g., Zeng and Takatsuka, 2009; Kung and Eslami, 2015). Few studies have compared group chats among speakers of English as a home language (EHL) and learners of English as a foreign language (EFL) and those among EFL speakers from different language backgrounds. Third, results are inconclusive concerning the effects of interlocutor types on FFEs. However, the language background of the partnering class is a critical decision for language instructors and students in establishing a collaborative online international learning (COIL) partnership.

Accordingly, this study investigated the effects of interlocutor types on errors and FFEs, both covert and overt, in text-based online group chats among Chinese and U.S. college students (EHL-EFL groups) and those among Chinese and Mexican college students (EFL-EFL groups). The students participated in meaning-focused communication for COIL projects with English as a shared language. The following questions were of particular interest:

RQ1. Did the two types of groups differ in the number of errors and feedback moves?

RQ2. What differences, if any, existed in the FFE characteristics between the two types of groups?

## Methods

### Instructional Settings

COIL aims to develop and implement team-taught courses with faculty members and university students from different lingua-cultural backgrounds (SUNY COIL Centre, 2015; O'Dowd, 2018). The main purpose is to expand international partnerships without costly physical mobility. COIL is generally offered at selective universities and by volunteer instructors without compensation. The two COIL partnerships in this study were established within the global network of the SUNY COIL Center (https://coil.suny.edu/) and intended to instill students with collaboration and global awareness, which are essential in autonomous learning with technology (Lewis, 2013; Lai, 2017). The instructors negotiated to find a common interest that suited the learning outcomes within a particular course designated for COIL and developed a shared syllabus. Language improvement was not a priority but a natural by-product (SUNY COIL Centre, 2015).

Both COIL projects in this study lasted for 7 weeks with a city-tourism-related topic. One was a partnership between an English course at a Chinese university and an art history course at a U.S. university (EHL-EFL project). Students explored the European-style buildings in the city of Tianjin. The other represented a collaboration between another cohort of the same English course and a cultural studies course at a Mexican university (EFL-EFL project). Students investigated the historical landmarks of a city in China, Mexico, or the U.S. These courses were not simultaneously scheduled due to time differences.

The two projects shared identical instructor-designed tasks and agendas (see Table 1). The tasks included information exchange (icebreaker 1), comparison and analysis (icebreaker 2), decision-making (research topic and research plan), and collaborative product creation (group presentation with slides) (O'Dowd and Ware, 2009; Canals, 2020). For example, in icebreaker 2, students compared the landmarks of their cities with text messages and pictures and submitted a summary of the comparison. During COIL, students could refer to the COIL syllabi for detailed task descriptions or ask the instructors for help via emails. The instructors also provided scaffolding in offline classes, which was based on students' queries raised in the classroom and emails. Most of the queries were related to procedural issues students encountered such as time difference, uneven participation among group members, and task requirements whereas language-related queries occurred only occasionally. The instructors intervened in some groups' projects when necessary. In this sense, COIL is a student-centered and teacher-guided learning method.

**Table 1:** General description of the COIL projects.

**Tasks**	**Descriptions**
Icebreaker 1	Familiarize each other in every international group with demographic information and campus life (Week 1)
Icebreaker 2	Compare the city landmarks on each side and make a summary of the comparison (Week 2)
Research plan and data collection	Decide on a group project topic around the COIL theme in consultation with the instructors (Week 3), design a research plan for data and academic source collection (Week 4), conduct their research, and prepare for a group presentation (Weeks 5 and 6)
Group presentation with slides	Summarize the research results and COIL experiences in a virtual conference (Week 7)

Students continuously communicated with their partners in text-based online chat groups to complete the aforementioned tasks. In the process, instant messengers such as WhatsApp and WeChat were used to generate a live-talk experience (see Table 2 for excerpts of the group chats). The communication could be casual or more structured in nature, depending on the tasks. Icebreaker 1 was relatively informal, engaging students in an introductory activity with text messages and pictures while other tasks were more structured, requiring students to communicate for the production of a particular product (O'Dowd and Ware, 2009). The group chats were self-organized without instructors' presence and occurred out of class. No side was instructed to pay special attention to language forms in COIL chats; students were reminded to be patient in case of communication breakdowns. Their communication was meaning-focused and information-oriented. Therefore, FFEs were supposed to be incidental in the chats.

**Table 2:** FFE coding framework.

**Characteristics**	**Categories and definitions**	**Examples**
Overtness	Overt: FFEs identified from chat logs alone	2C5: Does “makeup” means “cosmetics”
		M5: Yes, it means. (Icebreaker 1)
	Covert: FFEs understood or used by Chinese students with resources other than help in chats	2C5: Your portrait is so beautiful.
		M5: Thank u so much. I like this profile picture too.
		2C5: I want to change my portrait. (Checked “portrait” in a dictionary) (Icebreaker 1)
Type	Preemptive: unsolicited queries about linguistic items	See that for overt
	Reactive: error correction	1C2: There is 4 people speaking, correct?
		U2: Yes, there are four.
		Topic continued. (Preparation for the group presentation)
Linguistic focus	Grammatical	See that for reactive
	Lexical	See that for overt
	Mechanical	2C2: i think it would be very goog
		U5: good, sorry (Icebreaker 2)
Presence	Presence: feedback offered by another interlocutor after an error	See that for reactive
	Absence: no feedback offered by another interlocutor after an error	See that for mechanical level
Uptake	Uptake: response produced to the feedback	1C5: Also, it is Tianjin, not Tienjing
		U5: Oh sorry. Tianjin. (Preparation for the group presentation)
	No uptake: topic continuation after feedback	See that for reactive
Uptake quality	Successful uptake: incorporation of correct forms into subsequent production	See that for uptake
	Unsuccessful uptake: no incorporation of correct forms into subsequent production	See that for covert
Provider	Self-repair	See that for mechanical level
	Other-repair	See that for reactive
Source	Code: inaccurate use of forms with no apparent miscommunication	See that for reactive
	Message: intervention in meaning	See that for overt

### Participants

The combined classes were divided into groups. Each EHL-EFL group included four U.S. students and four to five Chinese students; each EFL-EFL group included three Mexicans and four Chinese. Groups instead of dyads were adopted to lower students' language and cultural anxieties and to secure a partnership in case of noncollaboration of some students. All international groups communicated as content learning peers and tried to build a symmetrical partnership.

To gain a more balanced comparison, chat logs of two EHL-EFL groups and four EFL-EFL groups were excluded either because they mainly communicated via emails or because only the local group leaders were involved in the cross-cultural communication. The subsequent data analysis included the data of five EHL-EFL groups (including 22 Chinese students and 20 U.S. students) and five EFL-EFL groups (including 20 Chinese students and 15 Mexican students).

All Chinese students (native Mandarin speakers) were first-year students of different majors. They all had learned English for about 10 years and were classified as advanced-level English learners based on scores of a placement test. The U.S. students were mostly junior or senior students majoring in design-related majors such as graphic design and technical design; two of them spoke home languages other than English, but they both claimed native-like English proficiency. All Mexican students (native Spanish speakers) were sophomores majoring in English teaching. According to the Common European Framework of Reference for Languages, their English proficiency was graded A2–B1. All participants gave consent to have their data analyzed for this study. Their personal information has been kept confidential.

### Instruments and Procedure

Every COIL group was instructed to submit their chat logs every week. The Chinese students were asked to recall immediately after a chat and mark on the chat logs (Microsoft Word files) with comments (in Chinese) indicating where and why they had corrected errors before sending out messages, consulted tools like dictionaries and online translation Apps, or sought help from their friends or teachers. These scenarios constituted covert FFEs. The recall only included linguistic items, not content. The Chinese instructor reminded students of this task every week and encouraged students to remind their local partners. A post-COIL questionnaire was distributed to all participants immediately after the COIL projects. Open-ended questions like “Have you noticed any language problems of your COIL partners during the chats” and “Did your partners' language errors affect your understanding? If yes, did you try to correct their errors? Why and why not?” were asked to probe students' attitudes toward FonF.

### Coding and Data Analysis

In this study, an overt FFE started either with an error that received feedback (reactive FFE) or with a query about a linguistic item (preemptive FFE). A covert FFE occurred when Chinese interlocutors noticed a gap either between “what they wanted to say” and “what they could say” or between “what they could understand” and “what other interlocutors said,” and then they resorted to resources other than help in chat groups to fill the gap. Identification of covert FFEs depended on Chinese students' stimulated recall. Errors were categorized in linguistic levels (spelling, vocabulary, and grammar) and interlocutor backgrounds (China, Mexico, and the U.S.).

Once FFEs were identified, they were coded for characteristics adapted from Loewen's (2010) framework (see Table 2 for the definitions, categories, and examples). Type, linguistic focus, presence of feedback, uptake, provider of repair move, and source were included, which have been found closely related to uptake quality (e.g., Loewen, 2005, 2010). Explicitness was excluded since almost all feedback was implicit in this study. Overtness was included, based on Smith's (2008; 2010) studies and on that overt FFEs cannot fully reflect FFEs in text-based online group chats.

All errors and FFEs were identified by two independent raters. The inter-rater reliability, based on percentage agreement, was 92.4% for error identification, 91.2% for linguistic levels of errors, and 98% for interlocutor backgrounds. The number of FFEs was small. Therefore, both raters coded all the chat logs, discussed the disagreement, and reached 100% agreement.

Raw frequencies and percentages for the categories were calculated. To compare the distribution of errors and FFE characteristics between the two types of groups, Pearson's chi-square analysis on the raw frequencies was performed with Statistical Package for the Social Sciences (SPSS) 27. Standardized frequencies were also calculated where necessary. In this study, raw frequencies worked better than standardized frequencies in reflecting how much effort students made to focus on form during the two COIL projects equal in length. The significance level was set at 0.05. Adjusted residuals greater than +2 or lower than −2 were applied to identify the source of significant differences.

## Results and Discussion

### RQ1: Differences of Errors and Feedback Between EHL-EFL Groups and EFL-EFL Groups

The Chi-square test showed a significant difference in the number of errors between the two types of groups (χ^2^ = 47.7, df = 1, *p* < 0.001) (see Table 3). The EHL-EFL groups committed more errors in absolute number (505 vs. 328) but produced a lower error rate (number of errors/K words) than the EFL-EFL groups (22.4 vs. 26.0). The Chinese students in the EHL-EFL groups committed much more errors than those in the EFL-EFL groups (420 vs. 178, i.e., 18.6/K words vs. 14.1/K words), especially vocabulary errors (100 vs. 29, i.e., 4.4/K words vs. 2.3/K words). This indicated that interaction with EHL speakers was more lexically taxing than that with EFL learners. Grammatical errors were more common than spelling and vocabulary errors in both types of groups (59.8 and 66.8%).

**Table 3:** Errors and feedback of EHL-EFL and EFL-EFL groups.

**Errors**	**EHL-EFL (22,569 words)**					**EFL-EFL (12,593 words)**				
	**China**	**U.S**.	**Total**	**Errors (/K words)**	**Feedback**	**Chinese**	**Mexico**	**Total**	**Errors (/K words)**	**Feedback**
	***n* (%)**	***n* (%)**	***n* (%)**		***n* (%)**	***n* (%)**	***n* (%)**	***n* (%)**		***n* (%)**
Spelling	65 (15.5)	28 (32.9)	93 (18.4)		2 (2.2)	26 (14.6)	42 (29.0)	69 (21.0)		3 (4.3)
Vocabulary	100^a^ (23.8)	10 (11.8)	110^a^ (21.8)		13 (11.8)	29^a^ (16.3)	9 (6.2)	40^a^ (12.2)		9 (22.5)
Grammar	255 (60.7)	47 (55.3)	302 (59.8)		3 (1.0)	123 (69.1)	94 (64.8)	219^a^ (66.8)		3 (1.4)
Total	420 (100)	85 (100)	505 (100)	22.4	18 (3.6)	178 (100)	145 (100)	328 (100)	26.0	15 (4.6)

a *Adjusted standardized residual>+2 or < −2*.

*For errors χ^2^ = 47.7, df = 1, p < 0.001*.

*For feedback χ^2^ = 1.8, df = 1, p = 0.174*.

Both types of groups demonstrated very low feedback rates (number of feedback moves/number of errors, 3.6 and 4.6%), and most errors went uncorrected. This corroborated the dominance of meaning over form in a communicative context (Zeng and Takatsuka, 2009; Kwon and Lee, 2011). The low feedback rates could be attributed to several reasons. First, information exchange rather than linguistic accuracy was the priority in COIL communication. Students might not have noticed errors (Bryfonski and Sanz, 2018; Peace, 2019). Second, pointing out others' errors might undermine social interaction, an important aspect of the sociality of autonomous learning (Lewis, 2013). Therefore, students might have refrained from offering feedback. Third, the instructors did not emphasize linguistic accuracy; otherwise, the EFL learners might hesitate to take risks in chats. The questionnaire results revealed that students seldom provided feedback unless the errors interfered with understanding. Instead, they deliberately avoided language repair. However, few corrections did not mean scant noticing of repairable items. For example, the Chinese students had a WeChat group with local peers only to offer each other feedback and to reflect upon their COIL chat performance. In COIL, students learned autonomously and created “relatedness needs” (Ryan, 1991, p. 210) for support with local and international peers. In this sense, COIL could help improve students' autonomy as a learner, as a language user, and as a person (Lai, 2017).

Vocabulary errors in both types of groups entailed a higher feedback rate (11.8 and 22.5%) than grammar and spelling errors, as lexical issues were more likely to interfere with understanding of the subject matter or the topic (Seedhouse, 1999). This was consistent with previous research findings (e.g., Choi and Iwashita, 2016; Dobao, 2016).

### RQ2: Differences of FFE Characteristics Between EHL-EFL Groups and EFL-EFL Groups

Table 4 presents frequencies and percentages of FFE characteristics. The EHL-EFL groups produced significantly more FFEs than the EFL-EFL groups (201 vs. 66, χ^2^ = 37.7, df = 1, *p* = 0.000; 8.9/K words vs. 5.2/K words). The former showed considerably more covert FFEs than the latter (164 vs. 28, i.e., 7.3/K words vs. 2.2/K words). It could be that Chinese students resorted to covert FFEs more when interacting with the U.S. students than with the Mexican students because the U.S. students' utterances were linguistically, especially lexically, more complex than the Mexican students' utterances. In this sense, EHL speakers could provide more opportunities than EFL learners for noticing language gaps.

**Table 4:** Characteristics of FFEs of EHL-EFL and EFL-EFL groups.

**FFE characteristics**		**EHL-EFL**	**EFL-EFL**	**Total**	**χ^2^**
		***n* (%)**	***n* (%)**	***n* (%)**	
Overt	Covert	164^a^ (81.6)	28^a^ (42.4)	192 (71.9)	37.7^***^
	Overt	37^a^ (18.4)	38^a^ (57.6)	75 (28.1)	
	Total	201 (100.0)	66 (100.0)	267 (100.0)	
Type^b^	Preemptive	4 (10.8)	7 (18.4)	11 (14.7)	0.9
	Reactive	33 (89.2)	31 (81.6)	64 (85.3)	
	Total	37 (100.0)	38 (100.0)	75 (100.0)	
Linguistic focus	Mechanical	11^a^ (5.5)	14^a^ (21.2)	25 (9.4)	
	Lexical	185 ^a^ (92.0)	47^a^ (71.2)	232 (86.9)	19.1^***^
	Grammatical	5 (2.5)	5 (7.6)	10 (3.7)	
	Total	201 (100.0)	66 (100.0)	267 (100.0)	
Source	Code	15^a^ (7.5)	18^a^ (27.3)	33 (12.4)	
	Message	186^a^ (92.5)	48^a^ (72.7)	234 (87.6)	18.0^***^
	Total	201 (100.0)	66 (100.0)	267 (100.0)	
Uptake^b^	No uptake	4 (20.0)	3 (15.0)	7 (17.5)	0.2
	Uptake	16 (80.0)	17 (85.0)	33 (82.5)	
	Total	20 (100.0)	20 (100.0)	40 (100.0)	
Uptake quality	Unsuccessful	9 (45.0)	11 (55.0)	20 (50.0)	0.4
	Successful	11 (55.0)	9 (45.0)	20 (50.0)	
	Total	20 (100.0)	20 (100.0)	40 (100.0)	
Provider	Self-repair	11 (55.0)	12 (60.0)	23 (57.5)	
	Other-repair	9 (45.0)	8 (40.0)	17 (42.5)	0.7
	Total	20 (100.0)	20 (100.0)	40 (100.0)	

a *Adjusted standardized residual>+2 or < -2*.

b *Only for overt FFEs*.

*** *p < 0.001*.

The two chat contexts produced a similar amount of overt FFEs in raw frequency (37 vs. 38), but the EFL-EFL groups produced almost twice as many overt FFEs as the EHL-EFL groups in terms of standardized frequencies (3/K words vs.1.6/K words). However, the EHL-EFL groups produced much more errors. This dovetailed that more proficient speakers could be more tolerant of errors that do not interfere with understanding (Ellis, 2013; Lightbown and Spada, 2013).

The number of covert FFEs was much larger than that of overt FFEs in the EHL-EFL project (164 vs. 37), but the distribution of covert and overt FFEs was more balanced in the EFL-EFL project (28 vs. 38). In the EHL-EFL project, students, as content learning peers, would rather not render the chats linguistically pedagogical or accentuate the discrepancy between their English levels. However, the EFL-EFL partnership was more linguistically symmetrical. It was less face-threatening and anxious to discuss language issues with EFL learning peers. In this sense, the EFL-EFL groups demonstrated greater willingness to focus on form overtly and higher sociality of language learning autonomy (Lai, 2017). Besides, the EFL-EFL chats did not contain many linguistically complex items that might entail covert FFEs, compared with the EHL-EFL chats.

The linguistic focus of FFEs differed significantly between the two types of groups (χ^2^ = 19.1, df = 2, *p* = 0.000), though lexical focus dominated in both (92.0 and 71.2%). The difference could be attributed to considerably more lexically-focused covert FFEs in the EHL-EFL chats than those in the EFL-EFL chats. Students explained in the questionnaire that lexical issues hampered comprehension and production more than mechanical and grammatical issues, and thus were easier to notice. Mechanical focus took up a small proportion, probably because mechanical errors generally did not cause miscommunication. However, it outweighed grammatical focus in frequency. Visual salience and easy recognition of mechanical errors pushed interlocutors to correct them (Crystal, 2001; Tudini, 2007).

The two types of groups showed a significant difference in the source of FFEs (χ^2^ = 18.0, df = 1, *p* = 0.000). The EHL-EFL groups produced significantly more FFEs interfering with understanding than the EFL-EFL groups, given that the former claimed more covert FFEs predominantly driven by communication needs. Meaning intervention, however, dominated both the EHL-EFL and the EFL-EFL chats (92.5 and 72.7%), which again reflected the meaning focus in COIL communication. Another point worth mentioning was that code (inaccurate language use with no apparent miscommunication) in the EFL-EFL chats (18, 27.3%) outweighed that in the EHL-EFL chats (15, 7.5%). This could be explained by previous research findings that language learners were more critical of language errors than native speakers in communication (Ellis, 2013; Lightbown and Spada, 2013), and by the fact that the Mexican students were English teaching majors, who had been trained to be conscious of errors.

Both types of groups showed a larger number of reactive than preemptive overt FFEs, but the small cell of preemptive FFEs in the EHL-EFL groups might affect the analysis. EFL learners did not intend to inquire international peers about linguistic forms as queries could reveal their linguistic inferiority (Loewen, 2010; Tudini, 2010), and COIL chats allowed extra processing time for covert FFEs. Also, COIL chats were information-oriented, and therefore students regarded queries about forms as inappropriate.

No significant differences were found in uptake, uptake quality, and repair providers. Following feedback, uptake was more likely to occur than topic continuation in both types of groups (80 and 85%). This was opposite to the findings by Mackey et al. (2003), possibly because of more processing time in text-based chats than that in face-to-face chats. Uptake can promote interlanguage development (Shekary and Tahririan, 2006) while covert FonF, which occurred frequently in this study, facilitated noticing gaps and arguably would benefit language learning too. The Chinese students reported that COIL communication constantly urged them to resort to online resources, from which they had learned many new expressions. Self-repair was preferred over other-repair in both projects (55.5 and 60.0%). The time delay in text-based chats could be a central factor for self-repair, which was less face-threatening than other-repair (Tudini, 2010).

## Conclusions

This study compared errors, feedback, and FFE characteristics in text-based online chats between the EHL-EFL groups and the EFL-EFL groups. The preference for meaning over form was obvious in both types of groups. Most of the errors went uncorrected, but uptake rates were high in both projects when feedback did occur. The EHL-EFL groups produced longer chats, more covert FFEs, higher lexical focus, and more miscommunication-induced FFEs than the EFL-EFL groups. However, both types of groups showed a preference for self-repair over other-repair and for lexical focus over mechanical and grammatical foci.

The implication of this research lies both in how to examine FFEs and in the design of COIL and other virtual exchange projects. The findings of covert and overt FFEs can shed light on how instructors could provide scaffolding effectively in COIL. Also, EFL instructors could use chat logs in offline focus-on-form instruction (Sun and Zhang, 2021). Besides, institution-based supports are crucial for the empowerment of students and instructors. For example, resources like teaching assistants, teacher training, and compensation can help with better teaching and learning.

Limitations still exist. First, covert FFEs were collected only from the Chinese students. Future studies might ask all students to recall covert FFEs for more nuances. Second, chat logs alone could not demonstrate acquisition. A longitudinal approach with tailored tests may be required to tap whether learners incorporate FFEs meaningfully into their interactional repertoires. Third, this study resembled a quasi-experimental design and therefore prevented a complete causal inference. Future studies might consider a more rigid experimental design.

## Data Availability Statement

The raw data supporting the conclusions of this article will be made available by the authors, without undue reservation.

## Ethics Statement

The studies involving human participants were reviewed and approved by Ethics Committee of Foreign Language College, Tianjin Normal University. The patients/participants provided their written informed consent to participate in this study.

## Author Contributions

RF and WZ conceived and designed the study, and analyzed the data. RF, KP, and RMF designed the two COIL projects and collected the data. RF drafted the manuscript. All authors revised the manuscript together and approved the version for publication.

## Funding

This work was supported by the Tianjin Philosophy and Social Science Research Project International Collaboration Community of Tertiary-level English Teaching-Learning-Research in Internet + era (Grant number: TJWW16-018).

## Conflict of Interest

The authors declare that the research was conducted in the absence of any commercial or financial relationships that could be construed as a potential conflict of interest.

## Publisher's Note

All claims expressed in this article are solely those of the authors and do not necessarily represent those of their affiliated organizations, or those of the publisher, the editors and the reviewers. Any product that may be evaluated in this article, or claim that may be made by its manufacturer, is not guaranteed or endorsed by the publisher.
